# The Physician, the Hospital, and the Community

**Published:** 1896-12-19

**Authors:** 


### The Physician, the Hospital, and the Community.

"The physician, if anyone, sees the world as it is." That is the deliberate judgment of Dr. Henry Dwight Chapin, one of the best known of the physicians of New York. In that all men will agree, at any rate to this extent, that that part of the world which the physician does actually see, he sees stripped of all artificial coverings, sees it naked and bare, and exactly as it is.

This capacity of the physician for seeing things as they are is of the greatest possible service to himself and to his patients. Dr. Chapin thinks it ought to be of much more service than it is to the hospital, to the medical profession in its corporate capacity, and to the community. "The physician's knowledge," we are assured, "can throw some helpful light on many of the questions of the day." And further, "by recognising and entering into a larger sphere the medical profession can take its true commanding position in the community at large." The farseeing doctor is able to say that the community must work its way towards the philosophical physician's standpoint, and see certain things with his eyes. If it does not, the community will go down, not up; will find degredation, not honour, in its advancing footsteps.

Dr. Dwight Chapin has a word for doctors; a word for hospitals; and a word for the community at large. To doctors he says, "the general practitioner is not yet in his decadence. He has a future of more importance than acting as a sort of intelligence officer to dole out his patients each to the proper specialist." There is obvious sense in that; and also in this, as general practitioners, specialists, and patients will do well to note: "It is too much the fashion for young graduates to start at once upon the exclusive practice of a specialty . . . they soon gain manual dexterity . . . but the skill is of the hand rather than of the head" . . . they remind us "more of the mechanic than of the physician" . . . "There seems to be a weakening of the judgment"; so that the specialist, though very competent sometimes to "remove an organ from the body," is the very last person in the world to be able to tell us honestly and judicially whether or not the organ really needs to be "removed." Now these are the weighty utterances of a mature physician of the very highest standing; and we repeat, and emphasise the repetition, that at this particular stage of medical progress, they demand the most serious and rational consideration at the hands of both doctors and patients.

Dr. Chapin tells hospital managers that one of the greatest evils associated with their work is its "segmental character." There is a "lack of proper conception and co-operation in the factors aiming at relief." . . . . What are urgently needed are "homes or retreats where poor convalescents can recuperate after their discharge from the hospital."

The average doctor thinks little of the general community; it is a name to him, no more. But at least he may pay the sort of attention to it which the farmer pays to his farm. Where should we all be without the community? How are we to make the community most serviceable to ourselves? By being most serviceable to it; and by insisting that when we are serviceable, it shall pay us for our services according to their intrinsic and sterling value. The services we render to the community are not temporarily or accidentally valuable as are those of the play-actor, the novelist, or even of the tailor, all of whom the world pays with great liberality. They are intrinsically and permanently valuable, and are indispensable. If we were wise we should cultivate the intelligence of the community from our own standpoint. Dr. Chapin tells us we must "broaden our professional ideals;" the "mere physician," he insists, must cease to be a "mere physician," and become a "mental and moral healer" in addition. "The old Egyptian idea of combining priest and physician" had much to say for itself. We ought to take for our sphere of duty the whole well-being of the whole of the people. Nothing less than this can satisfy the conscience of the really instructed physician.

